# Challenges in the Management of Silent Lactotroph Pituitary Adenoma

**DOI:** 10.1210/jcemcr/luaf241

**Published:** 2025-10-24

**Authors:** Melissa Hui Ting Leong, Yvette Li Yi Ang, Vincent Diong Weng Nga, Char Loo Tan, Jocelyn Yen Ling Wong, Doddabele Srinivasa Deepak

**Affiliations:** Division of Endocrinology, Department of Medicine, National University Hospital, National University Health System, Singapore 119074; Division of Endocrinology, Department of Medicine, National University Hospital, National University Health System, Singapore 119074; Division of Neurosurgery, Department of Surgery, National University Hospital, National University Health System, Singapore 119074; Department of Pathology, National University Hospital, National University Health System, Singapore 119074; Division of Neuroimaging, Department of Diagnostic Imaging, National University Hospital, National University Health System, Singapore 119074; Division of Endocrinology, Department of Medicine, National University Hospital, National University Health System, Singapore 119074

**Keywords:** silent lactotroph adenoma, pituitary neuroendocrine tumor, prolactinoma, pituitary magnetic resonance imaging

## Abstract

Silent lactotroph pituitary adenomas are rare tumors that clinically resemble nonfunctioning macroadenomas but are identified histologically by prolactin immunoreactivity. We report a 29-year-old woman with progressive visual loss caused by a large sella–suprasellar mass compressing the optic chiasm and invading the cavernous sinus. Initial endocrine evaluation was unremarkable, and magnetic resonance imaging (MRI) suggested a nonfunctioning adenoma. She underwent urgent surgical decompression, and histology confirmed a sparsely granulated lactotroph adenoma. Despite significant residual tumor on postoperative MRI, dopamine agonist therapy was not initiated due to absent dopamine receptor type 2 staining and patient-specific factors. Retrospective imaging review revealed atypical features, including heterogeneous T2-weighted signal, mixed cystic-solid morphology, and fluid-attenuated inversion recovery–T2 mismatch. This is potentially indicative of a silent subtype. This case underscores the diagnostic challenges posed by silent lactotroph adenomas and highlights the role of detailed imaging interpretation, histopathological analysis, and multidisciplinary evaluation in guiding management, especially in the absence of standardized treatment protocols for residual or recurrent disease.

## Introduction

Pituitary adenomas are a diverse group of anterior pituitary tumors with variable hormonal activity. Silent pituitary adenomas (SPA) present clinically as nonfunctional tumors but are found on histological examination to be immunoreactive for cell-specific transcription factors and express specific pituitary hormones. Silent lactotroph adenoma, representing approximately 0.6% to 1.65% of all pituitary adenomas, is 1 such SPA subtype [1]. This report presents such a case and reviews the relevant literature.

## Case Presentation

A 29-year-old woman was referred to the ophthalmology clinic for visual disturbance of 2 months' duration. She had no past medical history of significance and was working as a hair colorist in a salon. She reported difficulties with color perception and blurriness in her left eye (LE) over 3 months, which later progressed to involve her right eye (RE). She had reduced visual acuity in both eyes: LE could only detect hand movements while RE 6/45 vision on the Snellen chart. Additionally, a relative afferent pupillary defect was noted in her LE. She had a constricted visual field over her LE, while the RE revealed right inferior temporal quadrantanopia on the Humphrey visual field assessment.

## Diagnostic Assessment

**Figure 1.**
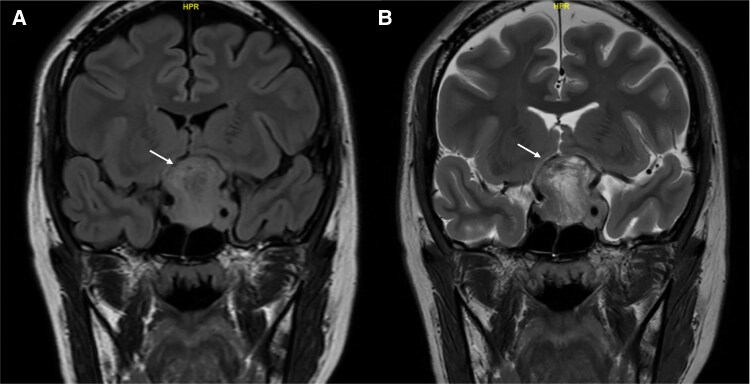
(A) Coronal section of unenhanced T2-weighted FLAIR MRI images of the brain preoperative. (B) Coronal section of unenhanced T2-weighted MRI images of the brain preoperative. Abbreviations: FLAIR, fluid-attenuated inversion recovery; MRI, magnetic resonance imaging.

Given these clinical findings, magnetic resonance imaging (MRI) of the brain and orbits was performed. It revealed a heterogeneously enhancing sella-suprasellar mass measuring 3.4 × 3.4 × 3.2 cm. This mass extended to the floor of the anterior cranial fossa, elevating and compressing the optic chiasm and part of the basifrontal lobes. There was an invasion of the left cavernous sinus with complete encasement of the left internal carotid artery (ICA), putting it at Knosp grade 4 (Fig. 1). Left optic nerve atrophy was also noted.

She was referred to neurosurgery and endocrinology for further evaluation. On review, she reported no symptoms suggestive of hormonal deficiency or excess. She attained menarche at the age of 15, had regular menstrual cycles since, and had no previous pregnancies. She was not on any medications and did not report any galactorrhea. A biochemical workup done at a different institution showed normal anterior pituitary hormones, including prolactin, suggesting a nonfunctioning pituitary macro-adenoma (Table 1). A dilution study of prolactin was not done at this stage.

**Table 1.** Pre- and postoperative pituitary hormone profile

Test name	Preoperative result	Postoperative result	Reference range
Serum cortisol (8:00 Am)	7.0 μg/dL (194 nmol/L)	7.5 μg/dL (208 nmol/L)	3.7-19.4 μg/dL (101-536 nmol/L)
TSH	1.69 μIU/mL (1.69 mIU/L)	2.03 μIU/mL (2.03 mIU/L)	0.35-4.95 μIU/mL (0.35-4.95 mIU/L)
Free T4	0.95 ng/dL (12.2 pmol/L)	0.84 ng/dL (10.8 pmol/L)	0.7-1.5 ng/dL (9.0-19.1 pmol/L)
LH	1.2 mIU/mL (1.2 IU/L)	4.1 mIU/mL (4.1 IU/L)	0.6-89.1 mIU/mL (0.6-89.1 IU/L)
FSH	5.4 mIU/mL (5.4 IU/L)	7.8 mIU/mL (7.8 IU/L)	1.4-16.7 mIU/mL (1.4-16.7 IU/L)
Estradiol	24.3 pg/mL (89 pmol/L)	30.3 pg/mL (111 pmol/L)	< 646 pg/mL (< 2382 pmol/L)
GH	0.19 ng/mL (0.19 μg/L)	0.56 ng/mL (0.56 μg/L)	0.13-9.88 ng/mL (0.13-9.88 μg/L)
IGF-1	118 ng/mL (118 μg/L)	186 ng/mL (186 μg/L)	115-292 ng/mL (115-292 μg/L)
Prolactin	12.5 ng/mL (264 mIU/L)	4.9 ng/mL (103 mIU/L)	2.3-19.1 ng/mL (73-407 mIU/L)

## Treatment

Due to the suspicion of a nonfunctioning pituitary macroadenoma associated with visual field and visual acuity compromise, she underwent a right pterional craniotomy and tumor excision. Intraoperatively, a moderately vascular tumor was found extending superiorly from the sella, displacing the optic chiasm and both optic nerves, and encasing the left ICA. Debulking was performed to decompress the suprasellar space, with adherent tumor remnants left on the underside of the optic chiasm and posterolateral to the left ICA. The remnant tumor shape is very irregular, and this makes estimation of volume difficult.

**Figure 2.**
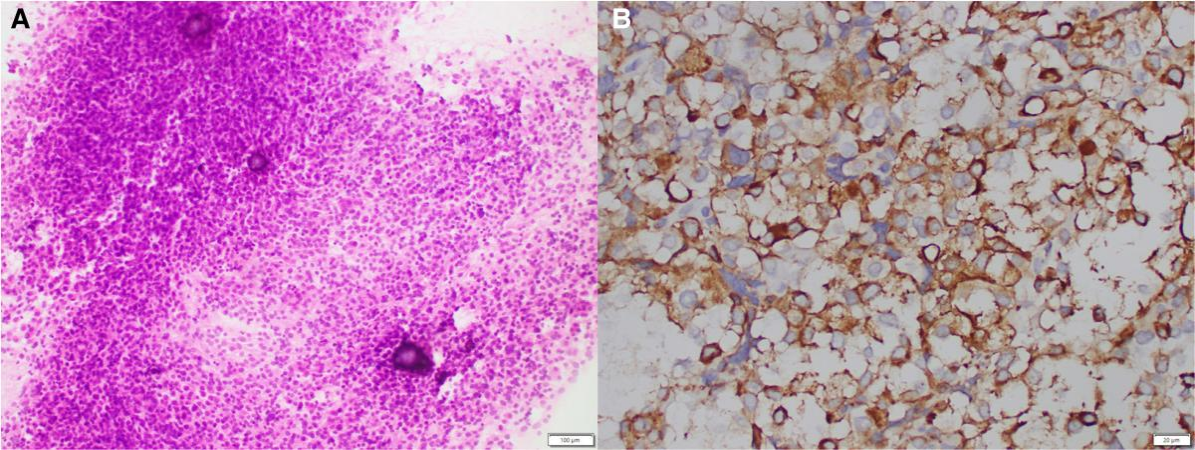
Histology staining of suprasellar tumor. (A) Intraoperative smear demonstrating cellular uniform tumor cells associated with scattered psammoma bodies. (B) Weak cytoplasmic and some paranuclear globular pattern seen with prolactin stain.

Histopathological analysis revealed tumor-composed polygonal cells with ample clear to chromophobic cytoplasm arranged in broad lobules and trabeculae associated with some psammomatous calcifications and spherules (Fig. 2A). Tumor cells showed diffuse positivity for synaptophysin, pituitary transcription factor 1, and somatostatin receptor type 2A. Prolactin stain showed diffuse, weak cytoplasmic and some paranuclear globular pattern (Fig. 2B). These indicate a silent lactotroph adenoma. Immunohistochemical stains for steroidogenic factor-1, T-box pituitary transcription factor, GATA-binding protein 3, ACTH, GH, TSH, and estrogen receptor (clone SP1) were all negative. Dopamine receptor type 2 (DR2) immunostaining was not performed due to resource limitations at the time of analysis. The Ki-67 proliferation index was less than 3%. In addition, there was an area of hemorrhagic necrosis, suggestive of tumor apoplexy. Histological diagnosis was of sparsely granulated lactotroph pituitary adenoma. Electron microscopy was not performed in this case.

In view of the unexpected histological finding of a lactotroph-secreting adenoma and significant residual tumor seen on postoperative MRI, prolactin was remeasured and found to be within normal range. A subsequent dilution study confirmed that the hook effect was not present.

Postoperatively, she had a complicated inpatient course and recovery. A reassessment of visual fields showed complete loss of vision in her LE and a temporal field defect in her RE with a visual acuity of 6/6. She was started on IV dexamethasone 8 mg twice a day to reduce postoperative optic nerve oedema. Five days after surgery, she developed a syndrome of inappropriate antidiuresis requiring IV hypertonic 3% saline, strict fluid restriction, and transient salt tablet supplementation to achieve normonatremia. Serum arginine vasopressin and copeptin were not measured directly. as the syndrome of inappropriate antidiuresis diagnosis was clinical. Dexamethasone was gradually tapered over 2 months, and at the dose of 1 mg once a day, this was changed to physiological hydrocortisone replacement (10 mg in the morning, 5 mg in the afternoon, and 5 mg in the evening). The patient also underwent inpatient rehabilitation for sensory ataxia, multidomain cognitive dysfunction, and apraxia.

## Outcome and Follow-up

**Figure 3.**
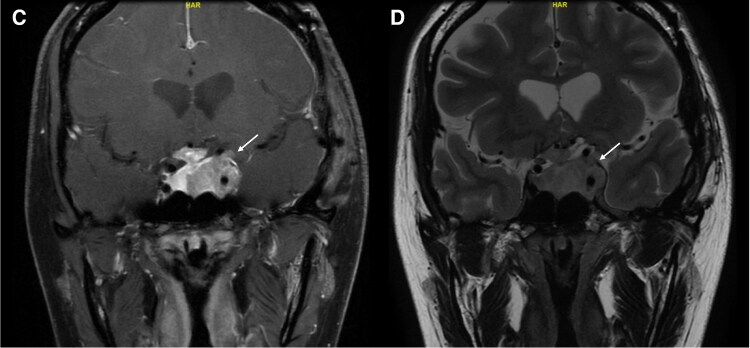
(C) Coronal section of T1-weighted postgadolinium MRI images of the brain postoperative. (D) Coronal section of T2-weighted MRI images of the brain postoperative. Abbreviation: MRI, magnetic resonance imaging.

At 4 months postoperative, she remained under multidisciplinary follow-up. Her vision was largely unchanged, with persistent LE blindness but improved color perception in the right. Her menstrual cycles were regular. Evaluation of her pituitary hormones showed that the 8 Am serum cortisol level was still low at 7.0 µg/dL (SI: 194 nmol/L) (reference range, 3.7-19.4 µg/dL [SI: 102-535 nmol/L]). We therefore opted not to undertake dynamic testing and continued on physiological hydrocortisone replacement with advice on steroid stress dosing during sickness. Her prolactin, thyroid, and GH levels remained within normal ranges. Although her estradiol levels were at the low-normal range, given regular menses, no hormone replacement therapy was needed. Follow-up MRI showed a significant residual tumor with a collapsed surgical cavity and mild left optic nerve displacement (Fig. 3).

## Discussion

SPA have been well-documented in the literature [2], and among these, silent lactotroph adenoma is a particularly intriguing subtype with low prevalence [3]. These tumors are characterized by their histological resemblance to prolactin-secreting tumors, yet they do not exhibit the associated hyperprolactinemia or its clinical manifestations.

Silent lactotroph adenoma differs from typical prolactin-secreting adenomas in terms of clinical presentation and biochemical profile [1]. The primary manifestation is a mass effect, with serum prolactin levels being either normal or mildly elevated, the latter due to the stalk effect. According to Drummond et al, silent lactotroph adenomas are more aggressive than typical prolactinomas and can behave more like nonfunctioning pituitary adenomas (NFPAs) in terms of mass effect and invasiveness [1]. Diagnostic certainty of a prolactinoma is generally considered when the serum prolactin concentration is >200 ng/mL (SI: >8700 mIU/L) [4]—in rare cases, up to 400 ng/mL (SI: ∼17,400 mIU/L) [5]. Differentiating silent lactotroph adenoma from other NFPA often requires specific immunohistochemical techniques to detect prolactin staining within tumor cells [6]. Historically, the 2017 World Health Organization classification identified silent lactotroph adenomas as part of the “high-risk” pituitary group, particularly in male patients, due to their propensity for aggressive behavior and higher recurrence risk [7]. Although our patient was female and presented with acute visual compromise due to mass effect, the sex discordance in tumor aggressiveness underscores the complexity of tumor behavior prediction and the need for individualized surveillance.

In our patient, visual symptoms prompted imaging, which revealed a sella–suprasellar mass without clinical or biochemical features of hyperprolactinemia. Preoperative prolactin dilution, thyrotropin-releasing hormone stimulation test, and dopamine agonist (DA) challenge were not performed due to normal prolactin levels, absence of galactorrhoea, and the need for urgent decompression. Additionally, the patient was not on any medications known to affect dopaminergic tone. Following the histology results, we performed serial dilution of a fresh postoperative blood sample, which did not show any hook effect despite a residual postoperative remnant tumor. Therefore, it was not a macroprolactinoma that could have been treated medically.

**Figure 4.**
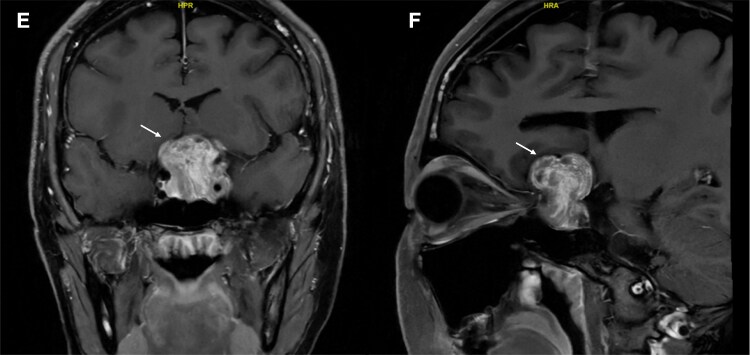
(E) Coronal section of T1-weighted postcontrast MRI images of the brain preoperative. (F) Sagittal section of T1-weighted postcontrast MRI images of the brain preoperative. Abbreviation: MRI, magnetic resonance imaging.

Recognizing silent lactotroph adenomas through imaging may prevent unnecessary surgeries. The T2 and fluid-attenuated inversion recovery coronal scans of the brain revealed typical features of a pituitary macroadenoma, including size >10 mm, a snowman configuration, and suprasellar extension (Fig. 1). In retrospect, the mass showed some atypical features, including a highly heterogeneous appearance on T2-weighted imaging with nodular and tubular T2 hyperintense cystic spaces, interspersed with T2 hypointense nodular and tubular structures, which was also evident on T1 postcontrast imaging (Fig. 4), with a heterogeneous mix of hypoenhancing and hyperenhancing areas. While MRI descriptions of silent lactotroph adenoma are limited, features such as multiple microcysts, cavernous sinus invasion, and size >40 mm have been associated with silent corticotroph pituitary adenoma [8]. Unique to this case was the highly heterogeneous appearance of mixed nodular and tubular T1/T2 hypo- and hyperintense structures, which were not reported previously. A fluid-attenuated inversion recovery-T2 mismatch sign was also observed, typically seen in certain adult brain glioma subtypes [9]. These distinctive imaging features suggest that the diagnosis extends beyond a typical nonfunctioning macroadenoma, indicating a more specific subtype.

Surgical intervention for NFPA, including silent lactotroph adenoma, is primarily indicated when the tumor causes significant mass effects on the optic apparatus. Silent lactotroph adenoma are typically diagnosed at a more advanced stage, making surgery more technically challenging due to the encasement of ICA, local invasion, and tumor fibrosis.

The potential role of DA in treating silent lactotroph adenoma is theoretical [10]. While DAs like bromocriptine and cabergoline are well-established in treating prolactinomas by targeting DR2 on lactotroph cells, their utility in silent lactotroph adenomas remains unclear. We explored the potential for off-label use of DA, given the persistent residual tumor mass. However, our laboratory had no access to staining for DR2 receptors in our patient's pituitary specimen to determine if a trial with DA therapy might be effective. Moreover, the patient volunteered information that she was prone to impulsivity and, given the association of the rare impulse control disorders with DA treatment [11], a mutual decision was made not to start DA. Given the lack of robust data, the use of these medications should be considered cautiously, balancing potential harms against the limited evidence of any benefit due to the rarity of these tumors.

In the event of tumor progression or recurrence, alternative management strategies may include repeat surgical resection or radiotherapy. Repeat surgery would be considered if the tumor growth results in symptomatic compression of nearby structures, such as the optic chiasm [12]. However, repeat surgery may have technical challenges due to tumor adhesion from scarring [13]. Radiotherapy, including stereotactic radiosurgery or fractionated radiotherapy, could be considered if the residual tumor shows signs of progression or cannot be safely resected further [14].

In conclusion, silent lactotroph adenomas are rare and often misdiagnosed due to their lack of overt hormonal activity. A high index of suspicion, careful histological evaluation, and multidisciplinary management are essential. While surgery remains the mainstay, the role of DA is uncertain and requires further study. Long-term surveillance is key to detecting recurrence and optimizing outcomes.

## Learning Points

- Silent lactotroph adenoma can mimic NFPA in both clinical presentation and biochemical profile.
- MRI features, such as heterogeneous T2 signal and cystic-solid architecture, may raise suspicion for a silent subtype; however, more data on lactotroph variants are needed.
- Routine preoperative dilution studies for prolactin are still important to avoid misdiagnosis due to the hook effect.
- Histological and immunohistochemical analyses, including prolactin and transcription factor staining, are essential for accurate tumor classification.
- Multidisciplinary evaluation and individualized decision-making remain key in managing rare and diagnostically challenging tumors such as silent lactotroph adenoma.

## Data Availability

Data sharing is not applicable to this article as no datasets were generated or analyzed during the current study.
